# Structural biomechanics determine spectral purity of bush-cricket calls

**DOI:** 10.1098/rsbl.2017.0573

**Published:** 2017-11-29

**Authors:** Benedict D. Chivers, Thorin Jonsson, Carl D. Soulsbury, Fernando Montealegre-Z

**Affiliations:** School of Life Sciences, University of Lincoln, Lincoln LN6 7DL, UK

**Keywords:** pure-tone, broadband, stridulation, path analysis, entropy, Orthoptera

## Abstract

Bush-crickets (Orthoptera: Tettigoniidae) generate sound using tegminal stridulation. Signalling effectiveness is affected by the widely varying acoustic parameters of temporal pattern, frequency and spectral purity (tonality). During stridulation, frequency multiplication occurs as a scraper on one wing scrapes across a file of sclerotized teeth on the other. The frequency with which these tooth–scraper interactions occur, along with radiating wing cell resonant properties, dictates both frequency and tonality in the call. Bush-cricket species produce calls ranging from resonant, tonal calls through to non-resonant, broadband signals. The differences are believed to result from differences in file tooth arrangement and wing radiators, but a systematic test of the structural causes of broadband or tonal calls is lacking. Using phylogenetically controlled structural equation models, we show that parameters of file tooth density and file length are the best-fitting predictors of tonality across 40 bush-cricket species. Features of file morphology constrain the production of spectrally pure signals, but systematic distribution of teeth alone does not explain pure-tone sound production in this family.

## 1. Introduction

Among insects, acoustic communication is common among the Orthoptera. Members of the suborder Ensifera (e.g. families: Tettigoniidae, Gryllidae, Prophalangopsidae) generate acoustic signals through tegminal stridulation, i.e. the rubbing together of their two modified forewings. One wing bears on its ventral surface a file of sclerotized teeth, while the opposite wing harbours a scraper (or plectrum) on its anal margin [1]. To generate the call, the scraper strikes the file and each tooth in series (a frequency multiplication mechanism), with the subsequent vibrations causing specialized wing cells to oscillate and radiate sound [2]. Each tooth strike generates a single oscillation (which decays in time), with the frequency at which any subsequent teeth are struck being the tooth-strike rate (TSR).

**Figure 1.**
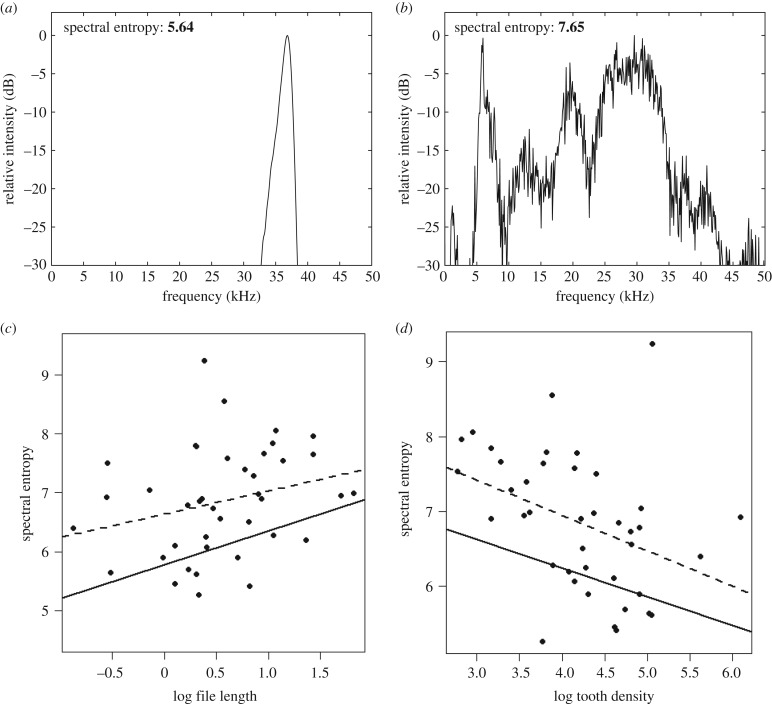
Example frequency spectra of (*a*) *Uchuca amacayaca* (pure-tone) and (*b*) *Panacanthus gibbosus* (broadband). Relationship between entropy and (*c*) file length and (*d*) tooth density. Linear regression line (solid line) and phylogenetically controlled (dashed line).

Across the approximately 7000 species of bush-crickets (Tettigoniidae), there is a considerable diversity of acoustic signals, with carrier frequencies ranging from the low audio (600 Hz) [3] to the extreme ultrasonic (approx. 150 kHz; [4]). They also incorporate widely varying levels of spectral purity (tonality), from highly resonant, pure-tone callers to broadband signals (figure 1*a,b*) [5–7]. The production of highly tonal signals is thought to require a consistent TSR during stridulation. In field crickets (Gryllinae), resonant, tonal sound production relies on an escapement mechanism that ensures a consistent frequency of energy input [8] and is reliant on the natural frequency of the radiating wing cells. This system is coupled with a complex phase shifter mechanism to ensure coherent sound radiation from two symmetrical wings [9]. In bush-crickets, which have a conspicuous morphological asymmetry between their wings, the escapement mechanism is not needed but wing resonance and file tooth arrangement are vital for resonant sound production where it occurs [5]. One hypothesis for bush-cricket frequency regulation combines morphology of the file with the mechanics of the wing during stridulation. A consistent TSR is attained by moving the scraper at a consistent velocity over systematically arranged teeth on the file, or alternatively, by moving a scraper at an increasing velocity across teeth that are spaced with correspondingly increasing distances [5,7]. This implies that for tonal production, the teeth should be organized systematically; otherwise tooth strikes will occur at varying time intervals, thus lowering spectral purity. In species that do not pass the scraper smoothly over a significant length of the file, e.g. extreme high-frequency callers [10] that use elastic energy to power the scraper, the distribution of teeth is less important and their arrangement more erratic.

An inherent problem in the analysis of non-resonant signals is the quantification, or statistical representation, of tonality. Traditionally, spectral purity has been quantified as the dimensionless index *Q* (high *Q* associated with highly tonal signals); however, this measure is not appropriate for non-resonant signals with asymmetrical spectra [11]. Recent work proposes spectral entropy (a measure of the complexity of a system) as a parameter of signal heterogeneity [12]. Herein, we use spectral entropy as an index for quantifying the varying scale of ordered, sinusoidal signals (tonal), to disorderly, towards random signals (broadband).

The sound generation structures of bush-crickets are not isolated units, meaning that changes in one morphological component may lead to concomitant changes in others. Understanding which of these components are important for producing the tonality or the frequency of the call is therefore challenging. We carried out a phylogenetic path analysis (PPA) across hypothesized paths through the sound generation units to investigate the role of variation in inter-tooth distance, among other factors, as a mechanistic driver of spectral purity.

## 2. Material and methods

### (a) Specimens and morphological measurements

Specimens of 40 species from 29 genera of neotropical bush-cricket were used. Species were chosen to represent a broad range of call types, from highly resonant to broadband singers and frequencies from approximately 5 to approximately 70 kHz (electronic supplementary material, table S1 and figure S1). Field-caught specimens were acoustically recorded and then preserved in alcohol. Digital photographs of the preserved files were taken on a scanning electron microscope (Inspect S50, FEI, Eindhoven, The Netherlands). Measurements of inter-tooth spacing were obtained using Coreldraw X4 (Corel Inc. 2005) (following established protocols described in [5]). From these measurements, file length, tooth width, file-bearing vein width, tooth density (tooth mm^−1^) and coefficient of variation of the inter-tooth distances (hereafter CV of tooth spacing) were calculated. Teeth of greatly varying distances at the extreme ends of the file were discounted from the measurements. Pronotal length was measured as a proxy of body size. File length, tooth density and pronotal length were log transformed for analysis.

### (b) Acoustic analysis

Wide-bandwidth, high-frequency microphones were used to record the calls for each specimen in laboratory conditions and the method of recording has been previously described [6]. Here, we quantify tonality as spectral entropy and this was obtained from a power spectral density (PSD, hanning window, nfft = 2048 points) over a representative syllable isolated from each species. Entropy *H* of the signal was calculated using the following equation:
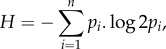
where *p_i_* is the probability mass function of the signal's PSD with length *n* [12,13]. Lower entropy values indicate towards pure tones and higher values towards random noise. To control for differences in the sampling frequencies of the different recordings, all acoustic data were resampled to 400 kHz prior to analysis. All acoustic analysis was carried out in Matlab (R2016b, The MathWorks Inc., Natick, MA, USA).

### (c) Phylogenetic path analysis

**Figure 2.**
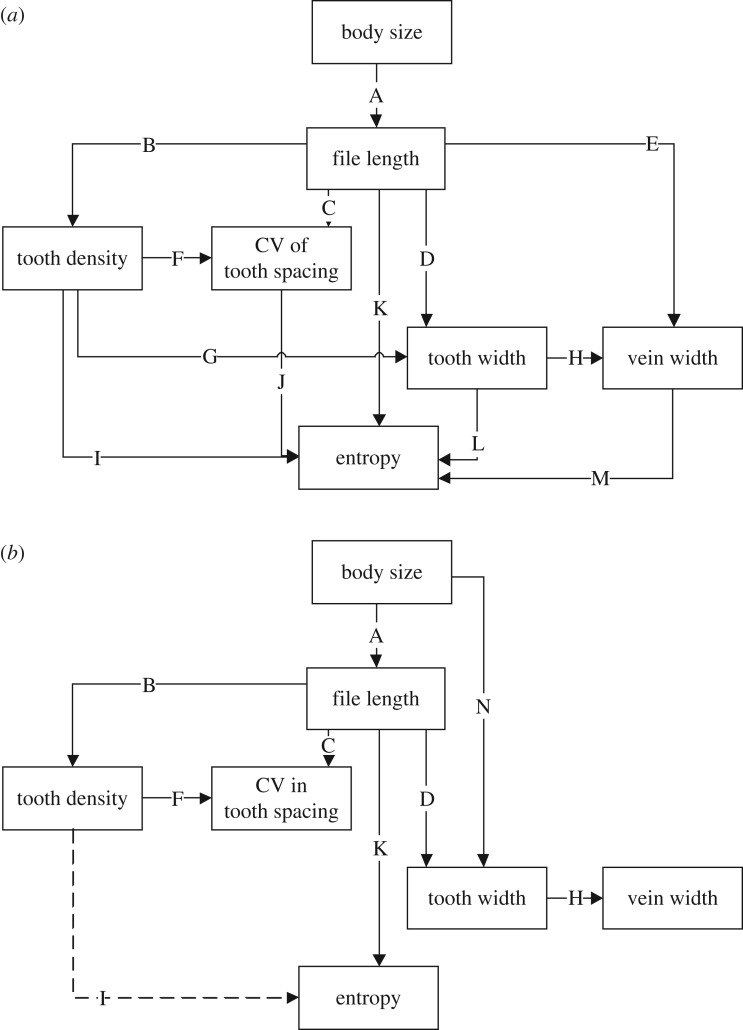
PPA for (*a*) global and (*b*) best-fitting hypothesized paths through morphological variables as predictors of entropy. Letters indicate individual paths, see tables 1 and 2.

Considerable interdependence exists in the morphological structure of bush-cricket sound generation units. To assess which features were most important in determining *H*, we used structural equation modelling (confirmatory path analysis: *sensu* [14]) to evaluate *a priori* piecewise structural equation models based on previous studies. We tested individual links between morphological components in an overall global path model (figure 2*a*). From the overall model, we used a backwards stepwise elimination process based on Akaike information criterion modified for small sample sizes (AICc) to remove non-significant pathways. In addition, we evaluated whether the non-hypothesized independent paths were significant and whether the models could be improved with the inclusion of any of the missing paths. As these data were not phylogenetically dependent, fitted models used a phylogenetic generalized least-squares regression (PGLS). The phylogeny of the 40 species used was adapted from the Tettigoniidae phylogeny presented by [15]. Models were run in R v. 3.2.1 [16] using the PGLS function from the caper package [17] and piecewise SEM analyses using the package piecewiseSEM [18].

**Table 1.** Structural equation model outputs for one proposed variable path (AICc = 104.74). See figure 2*a* for path diagrams.

path	variable	predictor	estimate ± s.e.	*p*-value
A	file length	body size	0.64 ± 0.25	0.013
B	tooth density	file length	−0.96 ± 0.14	<0.001
C	CV of tooth spacing	file length	7.06 ± 2.78	0.016
D	tooth width	file length	0.10 ± 0.04	0.011
E	vein width	file length	0.11 ± 0.06	0.067
F	CV of tooth spacing	tooth density	6.62 ± 2.16	0.004
G	tooth width	tooth density	−0.01 ± 0.03	0.631
H	vein width	tooth width	1.03 ± 0.32	0.003
I	entropy	tooth density	0.15 ± 0.32	0.645
J	entropy	CV of tooth spacing	−0.02 ± 0.02	0.289
K	entropy	file length	0.83 ± 0.45	0.067
L	entropy	tooth width	1.85 ± 1.89	0.335
M	entropy	vein width	−1.32 ± 0.88	0.147

missing paths	variable	predictor	estimate ± s.e.	*p*-value
	entropy	body size	−0.40 ± 0.48	0.407
	CV of tooth spacing	body size	−1.81 ± 3.42	0.600
	tooth width	body size	0.11 ± 0.04	0.017
	vein width	body size	−0.00 ± 0.09	0.966
	tooth density	body size	−0.44 ± 0.24	0.074
	tooth width	CV of tooth spacing	0.00 ± 0.02	0.468
	vein width	CV of tooth spacing	−0.01 ± 0.00	0.468
	vein width	tooth density	0.08 ± 0.05	0.176

**Table 2.** Structural equation model outputs for one proposed variable path. See figure 2*b* for path diagrams.

path	variable	predictor	estimate ± s.e.	*p*-value
A	file length	body size	0.64 ± 0.24	0.013
B	tooth density	file length	−0.96 ± 0.14	<0.001
C	CV of tooth spacing	file length	7.07 ± 2.78	0.016
D	tooth width	file length	0.09 ± 0.02	0.001
F	CV of tooth spacing	tooth density	6.62 ± 2.16	0.004
H	vein width	tooth width	1.40 ± 0.26	<0.001
K*	entropy	file length	0.58 ± 0.23	0.018
I*	entropy	tooth density	−0.38 ± 0.18	0.043
N	tooth width	body size	0.10 ± 0.04	0.014

missing paths	variable	predictor	estimate ± s.e.	*p*-value
	entropy	body size	−0.20 ± 0.41	0.624
	CV of tooth spacing	body size	−1.81 ± 3.41	0.600
	vein width	body size	0.01 ± 0.09	0.883
	tooth density	body size	−0.44 ± 0.24	0.074
	vein width	file length	0.11 ± 0.06	0.072
	tooth width	CV of tooth spacing	0.00 ± 0.00	0.313
	tooth width	tooth density	0.01 ± 0.03	0.804
	entropy	tooth width	0.70 ± 1.75	0.691
model B	entropy	file length	0.48 ± 0.35	0.186
	vein width	CV of tooth spacing	−0.01 ± 0.00	0.196
	entropy	CV of tooth spacing	−0.02 ± 0.02	0.466
model A	entropy	tooth density	−0.11 ± 0.27	0.685
	vein width	tooth density	0.07 ± 0.05	0.176
	entropy	vein width	−1.12 ± 0.83	0.185

## 3. Results

There was a significant interdependence of structural components of the sound generation unit (global model AICc = 104.74; table 1). Based on our predicted full model, only one excluded pathway (body size → tooth width) was significant, but there were multiple unsupported paths. There was a trend for file length to predict entropy (path K, *p* = 0.067; table 1).

Removal of non-significant pathways and inclusion of the one missing pathway significantly improved the model fit and left two plausible ‘best’ models (table 2 and figure 2*b*). In the first, there was a significant positive relationship between file length and entropy (model A: AICc = 74.091; table 2 and figure 1*c*). In the alternative pathway, there was a negative relationship between tooth density and entropy (model B: AICc = 75.01; table 2; figure 1*d*). CV of tooth spacing did not predict entropy (table 2).

## 4. Discussion

To our surprise, CV of tooth spacing did not predict entropy. Instead, the best-fitting models revealed an effect of file length or tooth density on entropy. Tooth density and CV of tooth spacing were not independent, so why is tooth density more important? Inter-tooth spacing increases along the file in the direction of scraper travel to provide room for the default increment in wing velocity [5,7]. This effect is also seen in certain species employing reverse stridulation, whereby the direction of scraper movement and increasing inter-tooth distances is reversed [19]. Hence, changes in inter-tooth distance can be compensated behaviourally [19].

Using PPA, we found that relatively shorter files produce more tonal calls, as do files of relatively higher tooth density. File length has been shown to scale negatively with tooth density [20], but these acted independently in our analysis. Shorter files, associated with species with smaller body size, produce higher frequency calls [20]. At higher frequencies of tooth strikes, maintaining a consistent TSR via any method of frequency regulation will have lower tolerances for variably time-shifted energy input that would result in lower tonal purity. Interestingly, certain bush-cricket species with relatively long files (e.g. species described in [3]) produce highly tonal calls at low frequencies (less than 5 kHz). This is suggestive of a differing form of frequency regulation at low frequencies, potentially an escapement mechanism similar to that observed in field crickets [8–9]. A higher density of teeth may impose a mechanical constraint through the possible height of each individual tooth (i.e. elevation of tooth cusps). Tonal calls originate in the correctly phased sequence of catch and release of the scraper during each tooth strike [21]. As the scraper passes over a rigid file, deeper teeth may cause disruption to this phasing, introducing variable catch and release sounds, thus lowering tonality. A consistent TSR may be facilitated by shallower teeth, whereby the phasing of both the catch and release is not time-shifted by variable catching of the scraper behind teeth of greater heights.

In conclusion, by analysing the inter-relationships between components of the bush-cricket sound generation unit, we demonstrate that file length and tooth density are the main factors driving tonal call production, and not variance in tooth spacing. The control of tooth-strike rate is likely to be critical to tonal call production, and both file length shortening and increasing tooth density are ways to ensure this.

## Supplementary Material

Supplementary figure S1

## Supplementary Material

Supplementary table S1

## Data accessibility

Data deposited in Dryad Digital Repository: http://dx.doi.org/10.5061/dryad.cb448 [22].
