# Supplementary material for: Structural biomechanics determine spectral purity of bush-cricket calls
Source: Biol Lett. 2017 Nov 29;13(11):20170573. doi: 10.1098/rsbl.2017.0573 (PMC5719386; doi:10.1098/rsbl.2017.0573)
Supplement: Supplementary table S1 [file rsbl20170573supp2.docx]

**Table S2.** All species and subfamilies with spectral entropy and carrier frequency.

| *Species* | *Subfamily* | *Frequency (kHz)* | *Spectral entropy* |
| --- | --- | --- | --- |
| *Acanthacara* nsp.1 | Conocephalinae | 21.1 | 6.90 |
| *Ancistrocercus excelsior* | Pseudophyllinae | 11.5 | 5.26 |
| *Artiotonus artius* | Conocephalinae | 40.8 | 5.69 |
| *Artiotonus captivus* | Conocephalinae | 41.6 | 6.85 |
| *Artiotonus tinae* | Conocephalinae | 35.7 | 6.74 |
| *Chibchella nigrospecula* | Pseudophyllinae | 9.8 | 7.66 |
| Cocconotus nsp.1 | Pseudophyllinae | 31.1 | 6.25 |
| *Copiphora gorgonensis* | Conocephalinae | 22.3 | 6.56 |
| *Copiphora gracilis* | Conocephalinae | 16.6 | 5.62 |
| *Daedalellus waehnerorum* | Conocephalinae | 34.8 | 6.79 |
| *Diacanthodis granosa* | Pseudophyllinae | 21.5 | 5.45 |
| *Eschatoceras* nsp.1 | Conocephalinae | 61.7 | 6.92 |
| *Eubliastes aethiops* | Pseudophyllinae | 22.3 | 6.28 |
| *Gnathoclita sodalis* | Pseudophyllinae | 16.6 | 6.51 |
| *Graminofolium* nsp.1 | Conocephalinae | 28.1 | 7.78 |
| *Ischnomella gracilis* | Pseudophyllinae | 15.2 | 5.90 |
| n. genus1 nsp.1 | Conocephalinae | 35.2 | 7.04 |
| *Myopophyllum* nsp.1 | Pseudophyllinae | 70.5 | 8.56 |
| *Panacanthus* nsp.1 | Conocephalinae | 7.3 | 6.99 |
| *Panacanthus cuspidatus* | Conocephalinae | 10.9 | 6.19 |
| *Panacanthus gibbosus* | Conocephalinae | 6.2 | 7.65 |
| *Panacanthus intensus* | Conocephalinae | 11.7 | 8.06 |
| *Panacanthus pallicornis* | Conocephalinae | 4.7 | 6.95 |
| *Panacanthus varius (Gorgona)* | Conocephalinae | 9.2 | 7.96 |
| *Panacanthus varius (Narino)* | Conocephalinae | 10.2 | 7.85 |
| *Panaploselis specularis* | Pseudophyllinae | 19.1 | 7.54 |
| nr. *Paranelytra* nsp.1 | Conocephalinae | 39.3 | 6.40 |
| *Parascopioricus cordillericus* | Pseudophyllinae | 27.0 | 7.39 |
| *Phlugis poecilla* | Meconematinae | 48.8 | 7.50 |
| *Phymonotus jacintotopus* | Tettigoniinae | 15.2 | 6.90 |
| *Ragoniella pulchella* | Conocephalinae | 22.9 | 7.58 |
| *Stetharasa exarmata* | Pseudophyllinae | 28.7 | 7.29 |
| nr. *Subria* nsp. 1 | Conocephalinae | 29.5 | 9.24 |
| nr. *Subria* nsp. 2 | Conocephalinae | 25.8 | 7.80 |
| *Teleutias fasciatus* | Pseudophyllinae | 22.7 | 6.11 |
| *Trichotettix pilosula* | Pseudophyllinae | 17.0 | 6.07 |
| *Triencentrus atrosignatus* | Pseudophyllinae | 23.8 | 6.98 |
| *Typophyllum mortuifolium* | Pterochrozinae | 9.0 | 5.41 |
| *Typophyllum trapeziforme* | Pterochrozinae | 22.1 | 5.89 |
| *Uchuca amacayaca* | Conocephalinae | 36.7 | 5.64 |
